# Signaling Mechanisms that Suppress the Cytostatic Actions of Rapamycin

**DOI:** 10.1371/journal.pone.0099927

**Published:** 2014-06-13

**Authors:** Stephan C. Jahn, Mary E. Law, Patrick E. Corsino, Bradley J. Davis, Jeffrey K. Harrison, Brian K. Law

**Affiliations:** 1 Department of Pharmacology and Therapeutics, University of Florida, Gainesville, Florida, United States of America; 2 University of Florida-Health Cancer Center, University of Florida, Gainesville, Florida, United States of America; Children's Hospital Boston & Harvard Medical School, United States of America

## Abstract

While rapamycin and the “rapalogs” Everolimus and Temsirolimus have been approved for clinical use in the treatment of a number of forms of cancer, they have not met overarching success. Some tumors are largely refractory to rapamycin treatment, with some even undergoing an increase in growth rates. However the mechanisms by which this occurs are largely unknown. The results presented here reveal novel cell-signaling mechanisms that may lead to this resistance. The absence of TGFβ signaling results in resistance to rapamycin. Additionally, we observed that treatment of some cancer cell lines with rapamycin and its analogs not only potentiates mitogenic signaling and proliferation induced by HGF, but also stimulates the pro-survival kinase Akt. Together, the data show that the effectiveness of rapamycin treatment can be influenced by a number of factors and bring to light potential biomarkers for the prediction of responsiveness to treatment, and suggest combination therapies to optimize rapalog anticancer efficacy.

## Introduction

Many human cancers have overactive mechanistic Target of Rapamycin Complex 1 (mTORC1), which contains mTOR, Raptor, and GβL, and functions as a protein kinase. Rapamycin and its analogs (rapalogs) are allosteric inhibitors of this complex and are approved by the Food and Drug Administration for use against mantle cell lymphoma [1], Estrogen Receptor positive breast cancers refractory to other treatments [2], as well as advanced metastatic renal cell carcinoma [3]. While they have proven effective in the treatment of these cancers, the rapalogs have not achieved widespread success as once hoped. The mTORC1 signaling pathway activates a negative feedback loop that involves the IGF1 receptor (IGF-1R), Insulin Receptor Substrate 1 (IRS1), and AKT, therefore inhibition of mTORC1 with rapalogs can activate this pathway [4], [5]. While this is known to be one mechanism of resistance to the cytostatic action of rapamycin and the rapalogs, most cases in which cancer cells are resistant to rapalogs are due to mechanisms that are currently not well understood.

Here we present new mechanisms that may explain tumor resistance to rapalogs and a new way in which mTORC1 signaling interfaces with cell cycle control. Previous studies indicated that rapamycin potentiates TGFβ-mediated cell cycle arrest [6]. Most nontransformed epithelial cells and a subset of carcinomas secrete TGFβ and respond to it in an autocrine manner [7]. We observe that ablation of TGFβ signaling in such cancer cell lines reduces rapamycin-induced arrest of proliferation, indicating that rapamycin effects are mediated in part through accentuation of TGFβ actions.

We also find that in some cancer cell lines rapamycin increases cell proliferation. One mechanism responsible for this is the potentiation of HGF/c-Met driven mitogenesis by mTORC1 inhibition. In other cancer lines such as the HCT116 colon cancer cell line, rapalogs and the mTORC1/2 inhibitor Torin increase tyrosine phosphorylation of a subset of cellular proteins and enhance the phosphorylation of proteins with Akt and PKC consensus phosphorylation sites. These effects parallel inhibitor-induced increases in the levels of IRS1, IGF-IRβ, phospho-Erk, and phospho-Akt[T308].

In summary, the data presented here provide new insights into mechanisms by which cancer responsiveness to rapamycin and rapalogs is determined, and these results may lead to future diagnostic analyses to predict which patients will benefit from these agents. Further, these observations suggest that rapalogs and c-Met inhibitors may function in a synergistic manner against some cancers. However loss of TGFβ signaling, as frequently occurs in human cancers, could suppress tumor responsiveness to mTORC1 inhibitors.

## Materials and Methods

### Cell Culture and Preparation of Lysates

Cell lines were maintained in Dulbecco's Modified Eagle's Medium supplemented with 10% fetal bovine serum in a humidified 37°C incubator with 5% CO_2_. Unless otherwise noted, cell lines were obtained from the American Type Culture Collection (ATCC) (Manassas, VA). The TβRII^flx/flx^ and TβRII^-/-^ hepatocyte cell lines were a gift from Dr. W. Grady [8], and the MMTV-PyMT,TβRII^flx/flx^ cell line was provided by Dr. H.L. Moses [9]. The neuT and neuT_EMT, CL2_ cell lines were previously described [10], [11]. Cell lysates were prepared as described previously [6].

### Cell Treatments

Compounds and growth factors used to treat cells were: Rapamycin, TGFβ, HGF (EMD Millipore, Billerica, MA), Activin (eBioscience, San Diego, CA), BMP4, Nodal, Torin1 (R&D Systems, Minneapolis, MN), Insulin, Transferrin, and Selenium (ITS) (Roche, San Francisco, CA), SU11274, AG490 (Sigma Aldrich, St. Louis, MO), and CGP57380 (Cayman Chemicals, Ann Arbor, MI). U0126 was obtained from Promega (Madison, WI). A TGFβ Type I Receptor kinase inhibitor (616451) was purchased from EMD Millipore (Billerica, MA).

### Construction of Stable Cell Lines

Lentiviral vectors used to construct the HCC1954 shScramble, HCC1954 shMet, and HCC1954 shBeclin cell lines were generated by co-transfecting shRNA constructs (Thermo Scientific, Waltham, MA) along with viral packaging plasmids pMD2.G and psPAX2 obtained from Addgene (Cambridge, MA) into the 293T cell line using Lipofectamine Reagent (Invitrogen, Grand Island, NY). Medium from the transfected 293T cell line was then used to infect the target cell line, which was subsequently selected using 5 µg/mL Puromycin. The MDA-MB-361/TPR-Met cell line was generated using the pBABE-puro TPR-Met retroviral vector (#10902) from Addgene.

### Immunoblots

Immunoblotting was performed as described [6], employing antibodies to phosphotyrosine (4G10, 05-321, EMD Millipore), PTEN (#9552), P-Smad 1/5/8 (#9511), P-Smad 3 (#9520), P-PKC[T514] (#9379), P-PKC[T638/641] (#9375), P-PKC[S660] (#9371), P-S6 (#2211), P-Akt[S473] (#9271), P-Akt[T308] (#9275), P-GSK3[S9] (#9331), and GSK3 (#9315) from Cell Signaling Technology (Danvers, MA) or IGF-1R (sc-713), and E2F4 (sc-866) from Santa Cruz Biotechnology (Santa Cruz, CA). All other antibodies used were purchased from commercial sources and listed previously [11]–[13].

### DNA-Pull Downs

Biotin-tagged oligonucleotides encoding wild type and mutant E2F promoter regions as described previously [14] were annealed to their complementary sequence by dissolving in annealing buffer (10 mM Tris, pH 7.5, 50 mM NaCl, 1 mM EDTA), heating to boiling, and allowing the oligonucleotides to slowly cool to room temperature, before coupling to streptavidin beads (Thermo Scientific, Waltham, MA). Double-stranded oligonucleotides bound to Streptavidin beads were then added to cell lysates and incubated for 2 hours at 4°C prior to washing and elution in SDS-PAGE sample buffer by boiling.

### Proliferation Assays

[^3^H]Thymidine incorporation assays were carried out as previously described [11], [15], using a two-hour [^3^H]Thymidine pulse.

### Statistical Analysis

All results presented were obtained in at least three independent experiments. Error bars represent the standard deviation of triplicate determinations. *P-*values were calculated using Student's *t*-test with GraphPad Prism, GraphPad Software, Inc., La Jolla, CA, and *P-*values less than 0.05 were considered statistically significant.

## Results

### Loss of TGFβ responsiveness as a factor in resistance to mTORC1 inhibitors

**Figure 1:**
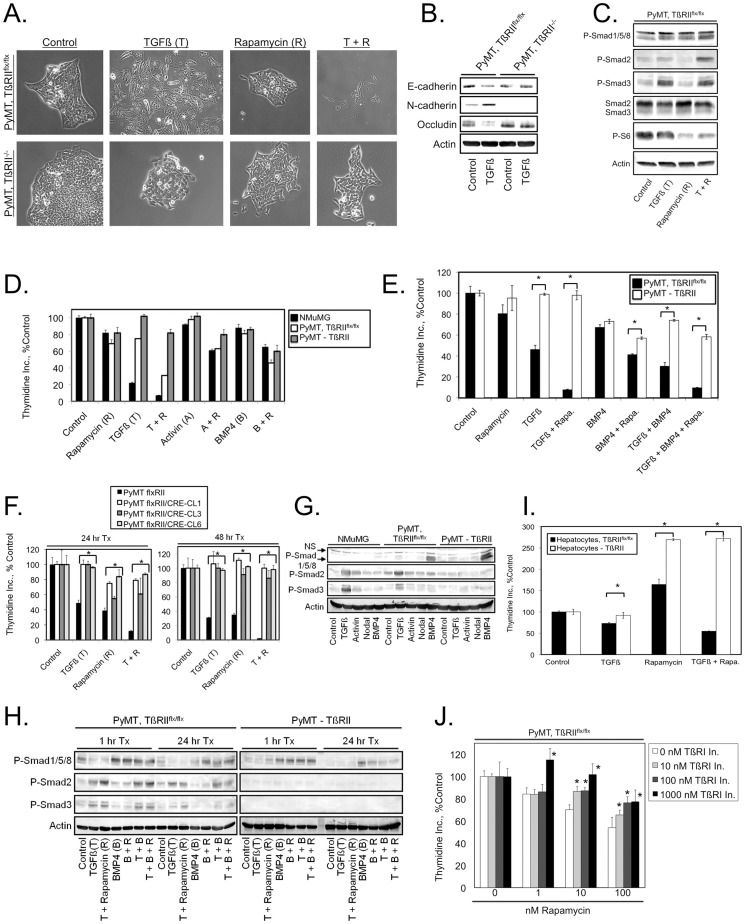
Rapamycin potentiates the growth inhibitory effects of TGFβ. (A) The indicated cell lines were treated with 2.5 ng/ml TGFβ, 100 nM rapamycin, or TGFβ + rapamycin (T + R) for one week until small colonies of cells formed. Representative images of treatment effects on cell-cell adhesion within colonies are presented. (B) The indicated cell lines were treated with or without 2.5 ng/ml TGFβ for 96 hours and cell extracts were analyzed by immunoblot. (C) PyMT, TβRII^flx/flx^ cells were treated for one hour with or without 2.5 ng/ml TGFβ and phosphorylation of Smads 2 and 3 and the ribosomal S6 protein were analyzed by immunoblot. (D–F) Proliferation, as measured by [^3^H]-thymidine incorporation, of the indicated cell lines after treatment for 24 hours [(D), (E), (F) left panel], or for 48 hours [(F) right panel]. (G, H) Immunoblot analysis of lysates from the indicated cell lines after 24 hours of the specified treatment for 24 hours (G) or either 1 or 24 hours (H). (I) Proliferation, as measured by [^3^H]-thymidine incorporation, of the indicated cell lines after 24 hours of the specified treatment. (J) PyMT, TβRII^flx/flx^ cells were treated with the indicated combinations of a TβRI kinase inhibitor and rapamycin at different concentrations for 24 hours. Asterisks indicate that the specified TβRI kinase inhibitor concentration reversed rapamycin-mediated cell cycle arrest in a statistically significant manner. Values represent the mean, and error bars are the standard deviation of triplicate measurements. * Denotes *P*<0.05 as determined using the unpaired Student's *t* test.

TGFβ signaling can produce pro- or anti-tumor effects depending on the context, and TGFβ signaling is frequently inactivated in human tumors through loss of expression of the TGFβ receptors or the Smad transcriptional regulators that mediate TGFβ signaling (reviewed in [16]). mTORC1 inhibitors such as the rapamycin analogs exhibit anticancer efficacy in some settings [17], and TGFβ and rapamycin cooperate to arrest the proliferation of a subset of cancer cells [6]. Thus, it is important to understand to what extent TGFβ signaling is required for mTORC1 inhibitors to produce their cytostatic effects. We examined the rapamycin sensitivity of matched cell lines derived from MMTV-PyMT mouse mammary tumors [18] with a functional TGFβ signaling pathway or that lacked TGFβ responsiveness due to Cre Recombinase-mediated deletion of the floxed gene encoding the TGFβ type II receptor (TβRII) [19]. Ablation of TβRII expression blocked TGFβ and TGFβ + rapamycin-induced loss of cell-cell adhesion, facilitating cell scattering (Fig. 1A), features associated with TGFβ-mediated epithelial to mesenchymal transition (EMT) [20]. Immunoblot analysis indicated that while TGFβ had no effect on EMT markers in the TβRII deficient PyMT cells, in the TGFβ signaling proficient PyMT cells TGFβ induced downregulation of the epithelial markers E-cadherin and Occludin and upregulation of the mesenchymal marker N-cadherin (Fig. 1B). As expected, TGFβ increased Smad3 phosphorylation in the PyMT, TβRII^flx/flx^ cells (Fig. 1C). Interestingly, TGFβ only weakly increased Smad2 phosphorylation in the PyMT, TβRII^flx/flx^ cells, but TGFβ-induced Smad2 phosphorylation was potentiated by co-treatment with rapamycin. Abrogation of TβRII expression using an adenovirus encoding Cre recombinase also decreased the ability of TGFβ, but not Activin or BMP4, to induce cell cycle arrest in a polyclonal cell population (Fig. 1D, 1E). Strikingly, in clonal cell lines lacking TβRII, cellular responses to TGFβ, rapamycin, and TGFβ + rapamycin were significantly blunted (Fig. 1F). Control experiments demonstrated that the absence of TβRII prevented TGFβ induction of Smad2 and Smad3 phosphorylation, but did not affect phosphorylation of Smads 1, 5, and 8 induced by BMP4 treatment (Fig. 1G, 1H). In previously described mouse hepatocyte cell lines with or without targeted TβRII deletion [8], TGFβ had little effect on proliferation, but rapamycin stimulated their proliferation (Fig. 1I). TGFβ + rapamycin treatment cooperated to suppress proliferation in the TGFβ responsive cell line, but resulted in the same increase in proliferation observed with rapamycin alone in the TβRII deleted cell line.

We collected conditioned medium from both cell lines and examined it for TGFβ activity using a cell line with a stably integrated TGFβ-responsive luciferase construct as described previously [10]. No TGFβ activity was detected in medium conditioned by either cell line suggesting that this particular cell line (MMTV-PyMT) is not regulated by autocrine TGFβ. However TGFβ is produced by a number of paracrine sources. TGFβ signaling is known to play an important role in the progression of MMTV-PyMT transgenic mouse mammary tumors [9], [21], [22].

If rapamycin cytostatic effects depend in part on potentiation of TGFβ responses then blockade of TGFβ receptor action with a kinase inhibitor should partially reverse rapamycin antiproliferative effects. Fig. 1J demonstrates that increasing concentrations of a TGFβ type I receptor kinase inhibitor partially reverse rapamycin antiproliferative effects over a range of rapamycin concentrations. Together, these observations suggest that depending on the status of TGFβ signaling, an important component of the rapamycin-mediated cell cycle arrest may result from potentiation of TGFβ cytostatic actions. Further, it is apparent that in some situations rapamycin may actually increase cell proliferation and that the net effect of rapamycin on the rate of cell division may depend significantly on the status of other pathways such as the TGFβ signaling axis.

### Potentiation of mitogenic signaling by rapamycin

**Figure 2:**
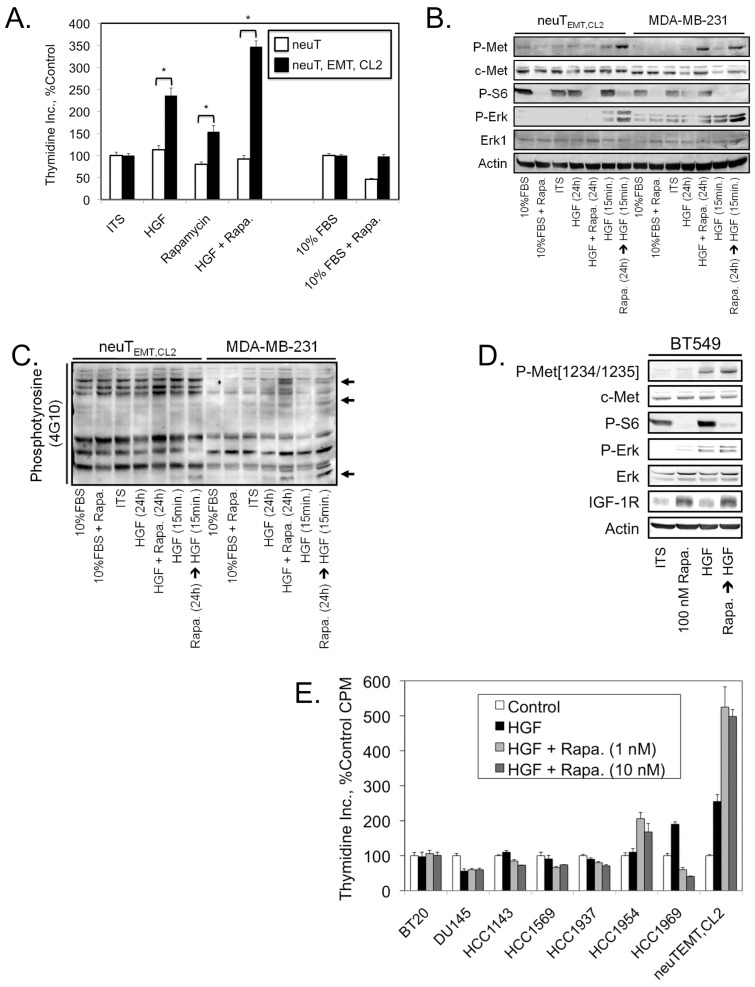
Rapamycin potentiates HGF-induced proliferation and mitogenic signaling in a subset of mammary carcinomas. (A) Proliferation, as measured by [^3^H]-thymidine incorporation, of the indicated cell lines after 24 hours of the specified treatments. Unless otherwise noted, HGF was used at 10 ng/ml and rapamycin was applied at 100 nM. Values represent the mean, and error bars are the standard deviation of triplicate measurements. * Denotes *P*<0.05 as determined using the unpaired Student's *t* test. (B) Immunoblot analysis of the indicated cell lines after the specified treatments. Unless otherwise noted, treatment intervals were 24 hours. (C) Immunoblot analysis of the indicated cell lines after the same treatments as in (B) using a pan-phosphotyrosine antibody. (D) Immunoblot analysis of BT549 cell lysates after the indicated treatments. HGF was added for the last 15 minutes of the 24-hour treatment period. (E) Proliferation, as measured by [^3^H]-thymidine incorporation, of the indicated cell lines after 24 hours of the specified treatments.

We previously showed that when an MMTV-Her2/neu cell line-derived mouse mammary tumor reaches approximately 1 cm in diameter the cells undergo an epithelial to mesenchymal transition (EMT), and that EMT is associated with dramatic changes in mitogenic signaling pathways and cell responsiveness to targeted therapeutics [23]. The post-EMT cells (neuT_EMT, CL2_) were found to express higher levels of the Hepatocyte Growth Factor (HGF) receptor c-Met. Therefore we examined the effect of rapamycin on the proliferation of neuT_EMT, CL2_ cells induced by HGF or Fetal Bovine Serum (FBS). Rapamycin increased the proliferation of the post-EMT cells in both the presence and absence of HGF when grown in medium without serum, but supplemented with Insulin, Transferrin, and Selenium (ITS) (Fig. 2A). However, the proliferation of neuT_EMT, CL2_ cells was not significantly altered by rapamycin when grown in medium containing 10% FBS. The rate of division of the pre-EMT cells did not change in response to HGF or rapamycin administered either alone or in combination, and was inhibited by rapamycin when 10% FBS was employed as the mitogen. Immunoblot analyses were performed on extracts from neuT_EMT, CL2_ cells and the MDA-MB-231 human breast cancer cell line to examine the cross-talk between HGF/c-Met and mTORC1 signaling (Fig. 2B). In both cell lines rapamycin potentiated HGF-dependent induction of c-Met and Erk1/2 phosphorylation and this effect was particularly prominent if the cells were pretreated with rapamycin for 24 hours, followed by a 15 minute stimulation with HGF. Examination of total levels of protein tyrosine phosphorylation showed several proteins that were most prominently phosphorylated in response to combined HGF and rapamycin treatment (Fig. 2C, arrows).

**Figure 3:**
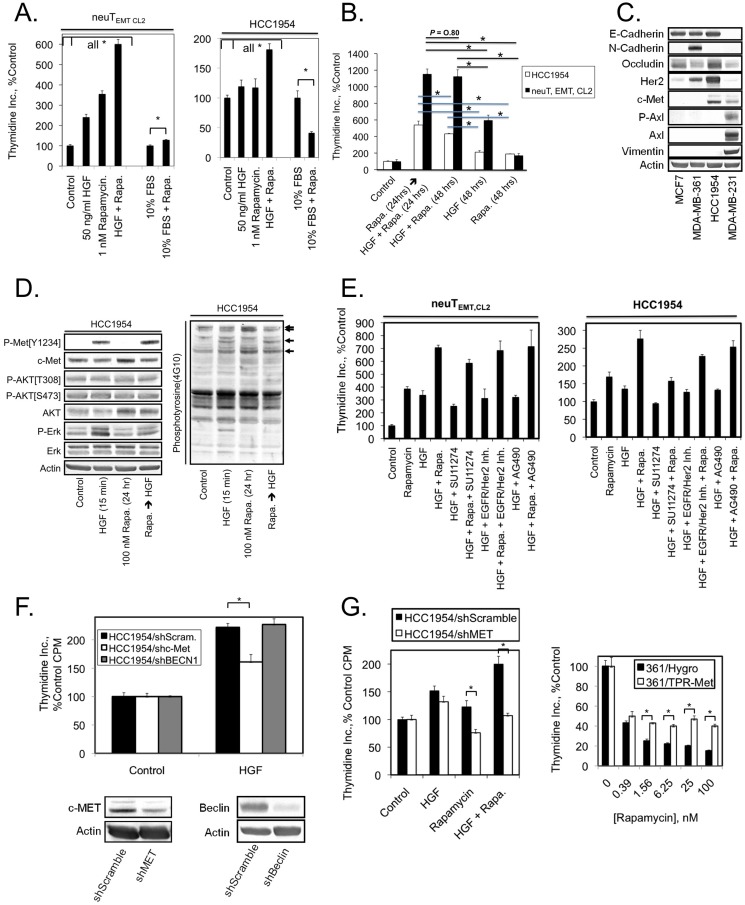
c-Met is required for rapamycin potentiation of HGF-mediated cell division. (A) Proliferation, as measured by [^3^H]-thymidine incorporation, of the indicated cell lines after 24 hours of the specified treatments. (B) Thymidine incorporation study carried out as in Fig. 3A, to compare the effect of rapamycin pretreatment for 24 hours followed by treatment with rapamycin + HGF for 24 hours versus co-treatment with rapamycin + HGF for 48 hours. Black lines represent comparisons between HCC1954 samples and blue lines are comparisons between neuT, EMT, CL2 samples. Asterisks correspond to differences with *P*<0.05. (C) Immunoblot analysis of the indicated cell lines. (D) Immunoblot analysis of HCC1954 cells after the indicated treatments. (E–G) Proliferation, as measured by [^3^H]-thymidine incorporation, of the indicated cell lines after 24 hours of the specified treatments. Panel F also shows an immunoblot analysis of c-MET and Beclin knockdown efficiency in the respective cell lines. Values represent the mean, and error bars are the standard deviation of triplicate measurements. * Denotes *P*<0.05 as determined using the unpaired Student's *t* test.

Similar, albeit less pronounced results were obtained with BT549 breast cancer cells (Fig. 2D), but under these conditions, neither HGF, rapamycin, nor HGF + rapamycin had significant effects on BT549 cell proliferation. Therefore, we screened a small panel of human cancer cell lines to determine whether rapamycin might potentiate HGF-mediated mitogenesis of some human tumors (Fig. 2E). Of these cell lines, only HCC1954 human breast cancer cells exhibited responses similar to neuT_EMT, CL2_ cells, although the proliferative responses were lower in magnitude. Further examination of the properties of HCC1954 cells demonstrated that their proliferation was weakly stimulated by both HGF and rapamycin, but more strongly increased by combined treatment with HGF + rapamycin (Fig. 3A, right panel). To examine whether rapamycin potentiation of HGF-driven proliferation requires the presence of HGF for the entire 48-hour treatment window, or produces the same response if added only during the final 24 hours, additional cell proliferation assays were performed. With neuT_EMT, CL2_ cells the rate of proliferation was the same whether HGF was present during the entire 48 hours or only during the final 24 hours of the treatment period (Fig. 3B). With HCC1954 cells the rate of proliferation was only slightly higher if HGF was only present during the last 24 hours of the treatment period, but the difference was statistically significant. The HCC1954 cells express relatively high levels of the receptor tyrosine kinases c-Met and Her2 and the epithelial markers E-cadherin and Occludin, but lack the mesenchymal markers Vimentin, Axl, and N-cadherin (Fig. 3C). These observations suggest that the ability of rapamycin to potentiate HGF/c-Met-mediated proliferation is not related to the mesenchymal versus epithelial phenotype of the particular cancer cell line.

We next performed immunoblot analyses of HCC1954 extracts (Fig. 3D). HGF increased c-Met tyrosine phosphorylation, but rapamycin only weakly potentiated this response to HGF, and rapamycin actually suppressed HGF-induced Erk phosphorylation. Examination of the overall tyrosine phosphorylation patterns in the HCC1954 cells indicated that the mechanisms of cross talk between HGF/c-Met and mTORC1 signaling pathways is likely to be complicated. Tyrosine phosphorylation of some proteins was increased by either HGF or rapamycin alone, and in other cases rapamycin blunted HGF-mediated tyrosine phosphorylation events. We performed additional studies using pharmacological and genetic approaches to verify that the pro-proliferative effects of rapamycin on neuT_EMT, CL2_ and HCC1954 cells are dependent on HGF/c-Met signaling. The c-Met inhibitor SU11274, but not inhibitors of the Epidermal Growth Factor Receptor (EGFR) or both EGFR and Her2, reduced the proliferative effects of HGF and rapamycin on HCC1954 cells (Fig. 3E).

Partial knockdown of c-Met decreased the ability of HGF to drive the proliferation of HCC1954 cells, but knockdown of the autophagy protein Beclin 1 was without effect (Fig. 3F). Further, c-Met knockdown blunted HGF + rapamycin-induced proliferation (Fig. 3G, left panel). These experiments indicated that c-Met is required for rapamycin potentiation of the proliferation of neuT_EMT, CL2_ and HCC1954 cells, but did not determine whether c-Met expression is sufficient for this effect. This issue was addressed by expressing the constitutively activated mutant of c-Met, TPR-Met [24], in the MDA-MB-361 cells that do not express c-Met [23], [25] and are moderately responsive to rapamycin antiproliferative effects [12]. TPR-Met expression partially reversed rapamycin cytostatic actions across a broad range of rapamycin concentrations (Fig. 3G, right panel). The results in Figs. 2 and 3 indicate that in a subset of cell lines that express c-Met, rapamycin potentiates HGF/c-Met-mediated proliferation and that this can either reduce the cytostatic effects of rapamycin observed, or in extreme cases rapamycin can increase proliferation.

### mTORC1/2 inhibitors stimulate Akt phosphorylation in HCT116 cells

One of the first mechanisms implicated in cancer resistance to mTORC1 inhibitors involves the inactivation of negative feedback loops by mTORC1 inhibitors, resulting in the inadvertent phosphorylation and activation of Akt [4]. Akt activation involves phosphorylation of Thr^308^ by phosphoinositide dependent kinase 1 (PDK1) [26] and phosphorylation of Ser^473^ primarily by mTORC2 [27], [28]. Thus, one rationale for the development of mTORC1/2 ATP-competitive inhibitors was to block mTORC1 signaling without simultaneously activating Akt. However, comparing the effects of rapalogs and mTORC1/2 inhibitors such as Torin can be complicated by the fact that prolonged rapamycin treatment can in some cases inhibit mTORC2 [29], and Ser^473^ can also be phosphorylated by other kinases (reviewed in [30]). Rapamycin resistance may also arise at the level of 4EBP1 that can be phosphorylated by either mTORC1 or MNK1 in a redundant manner [31].

**Figure 4:**
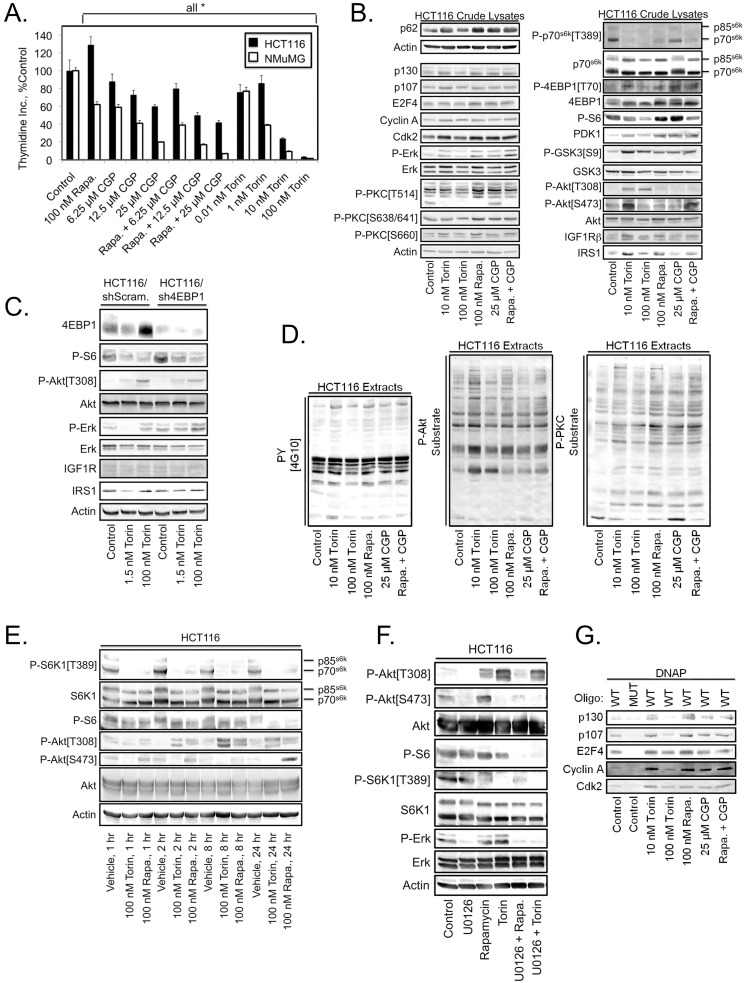
mTORC1/2 inhibitors potentiate mitogenic signaling in rapalog resistant HCT116 cells. (A) Proliferation, as measured by [^3^H]-thymidine incorporation, of the indicated cell lines after 24 hours of the specified treatments. Values represent the mean, and error bars are the standard deviation of triplicate measurements. * Denotes *P*<0.05 as determined using the unpaired Student's t test. (B) Immunoblot analysis of HCT116 cell lysates after 24 hours of the indicated treatments. (C) Immunoblot analysis of lysates from HCT116 cells stably transduced with control (shScramble) or sh4EBP1 constructs. Cells underwent the indicated drug treatments for 24 hours. (D) Immunoblot analysis of HCT116 cell lysates after 24 hours of the indicated treatments with antibodies recognizing tyrosine phosphorylated proteins (left panel), proteins phosphorylated on a consensus Akt substrate sequence (center panel), or a consensus PKC substrate motif (right panel). (E) HCT116 cells were treated for the indicated time periods with 100 nM Torin or 100 nM rapamycin and analyzed by immunoblot. (F) HCT116 cells were treated for 24 hours with 10 µM U0126, 100 nM rapamycin, 100 nM Torin, or the indicated drug combinations and analyzed by immunoblot. (G) Immunoblot analysis of E2F1 promoter DNA oligonucleotide pulldowns from HCT116 cell lysates after 24 hours of the indicated treatments.

The proliferation of HCT116 colon cancer cells is either not suppressed by mTORC1 inhibition, or is weakly stimulated. Therefore we examined whether the proliferation of HCT116 cells and NMuMG non-transformed mouse mammary epithelial cells could be blocked either by combining rapamycin with the MNK1 inhibitor CGP57380, or by incubation with the mTORC1/2 inhibitor Torin (Fig. 4A). CGP57380 caused a concentration-dependent decrease in the proliferation of both cell lines, but did not strongly potentiate rapamycin effects. In contrast to rapamycin, Torin potently decreased the rate of division of both cell lines. Immunoblot analysis of HCT116 extracts showed that Torin more completely blocked p70^s6k^ and 4EBP1 phosphorylation than rapamycin. Interestingly, Torin increased Akt phosphorylation on Thr^308^ and increased phosphorylation of the Akt substrate Glycogen Synthase Kinase 3 (GSK3) (Fig. 4B). Low concentrations of Torin (10 nM) increased expression of Insulin-like Growth Factor Receptor 1β (IGF1Rβ) and Insulin Receptor Substrate 1 (IRS1), but higher concentrations (100 nM) did not, suggesting that the increased phosphorylation of Akt on Thr^308^ was independent of Torin effects on IGF1Rβ and IRS1 levels. However the biphasic increase in Akt Ser^473^ phosphorylation at 10 nM Torin, but decrease at 100 nM Torin may result from the IGF-IR and IRS1 upregulation observed at the lower Torin concentration. Rapamycin effects on Thr^308^ phosphorylation were weaker, suggesting that mTORC2 inhibition has a role in Torin-induced increases in Akt phosphorylation on Thr^308^. Alternately, it is possible that this effect requires more complete inhibition of mTORC1 than can be affected by rapamycin. Akt phosphorylation on Thr^308^ was not downstream of 4EBP1 phosphorylation because it was not blocked by 4EBP1 knockdown (Fig. 4C).

Immunoblot analyses employing antibodies recognizing proteins phosphorylated on tyrosine, or proteins phosphorylated on Akt or PKC consensus recognition sequences showed that the tyrosine phosphorylation of a subset of proteins is increased by mTOR inhibitors (Fig. 4D, left panel). Interestingly rapamycin and Torin both increased the phosphorylation of multiple proteins on Akt and PKC consensus sequences (Fig. 4D, middle and right panels). These results suggest that the HCT116 cells are partially resistant to low concentrations of mTORC1 and mTORC1/2 inhibitors because of their ability to activate mitogenic signaling pathways. However it is also possible that other signaling pathways have become activated in HCT116 cells that are redundant with the mTORC1 pathway, rendering the cells refractory to rapalogs. To further examine the effects of rapamycin and Torin on Akt phosphorylation, time course experiments were performed. These studies indicated that while both Torin and rapamycin strongly decreased S6K1 phosphorylation on Thr^389^ (Fig. 4E), this did not correlate with loss of S6 phosphorylation at the earlier time points, suggesting the presence of an S6K1-independent mechanism of S6 phosphorylation in HCT116 cells. Torin, and to a lesser extent rapamycin, increased Akt phosphorylation on Thr^308^ at the 2, 8, and 24 hour time points, while 100 nM Torin blocked Akt phosphorylation on Ser^473^ at all time points examined. To assess whether the MEK/Erk pathway might be the pathway that renders S6 phosphorylation insensitive to inhibition by rapamycin, we examined whether the MEK inhibitor U0126 altered S6 phosphorylation either alone or in combination with rapamycin or Torin. Interestingly, S6 phosphorylation was only blocked by combined treatment with U0126 + rapamycin or U0126 + Torin, but was not significantly decreased by any of these agents when applied alone (Fig. 4F). U0126 unexpectedly blocked basal Akt phosphorylation on Ser^473^. U0126 also partially suppressed Torin- and rapamycin-induced phosphorylation of Akt on Thr^308^. Together, the results in Fig. 4A–F demonstrate that significant cross-talk and functional redundancy exist between the Akt/mTOR and MEK/Erk axes in HCT116 cells that may account for the ability of these cells to resist rapamycin-mediated cell cycle arrest.

Rapamycin inhibition of cell proliferation was shown to occur through suppression of E2F-dependent transcription [32], and in the case of TGFβ + rapamycin cytostatic effects involve loss of Cdk2 association with E2F4- and p107- or p130-containing complexes [6]. Therefore, we next examined the effects of low (10 nM) and high (100 nM) concentrations of Torin as compared with 100 nM rapamycin on E2F DNA binding complexes (Fig. 4G). Strikingly, 10 nM, but not 100 nM Torin, increased Cdk2 association with the E2F1 promoter element. The same result was obtained with 100 nM rapamycin, and in both cases increased binding of Cdk2 to the complex correlated with a dramatic increase in the levels of Cyclin A in the complex. The observations at the level of E2F4/Cdk2 DNA binding complexes again support the contention that the effects of Torin treatment are highly dependent on the concentration used and can cause opposite effects at different concentrations.

These results likely have important implications for mTORC1/2-targeted therapy. First, the findings indicate that the effects of mTORC1/2 inhibitors may in some cases be strongly concentration dependent with low and high concentrations producing differing responses. Second, in some cell lines, mTORC1/2 inhibitors increase phosphorylation of Akt on Thr^308^.

## Discussion

Rapamycin and the rapalogs have found a number of uses in the clinic. mTORC1 inhibitors are utilized not only in anti-cancer therapy, but also in anti-rejection therapy for transplant patients as well as in a coating on stents in order to prevent restenosis. In all of their uses, the antiproliferative effects of these drugs contribute to their clinical efficacy. However, these interesting compounds have yielded inconsistent results, leading to the need to better understand the molecular mechanisms by which they block cell division. The results reported here indicate that cross talk exists between the mTORC1 and TGFβ signaling pathways that might influence the therapeutic efficacy of mTORC1 inhibitors against specific tumors.

In some situations, rapamycin potentiation of TGFβ-mediated cell cycle arrest may play an important role in the cytostatic effects of mTORC1 inhibitors. The magnitude of this effect will depend on whether the cell type involved undergoes TGFβ-induced cytostasis. Some mesenchymal cell types have intact TGFβ signaling pathways and mount transcriptional responses to TGFβ, but TGFβ does not significantly affect their proliferation. Further, many cancer cells lack expression of the TGFβ receptors or the TGFβ-activated transcriptional regulators Smads 2, 3, and 4. In addition, the levels of autocrine or paracrine TGFβ to which cells are exposed, or the levels of basal ligand-independent TGFβ signaling may vary. The MMTV-PyMT transgenic mice provide a useful model of human breast cancer because the steps in their progression are similar to those of human tumors [18]. With MMTV-PyMT cancer cells rapamycin more effectively inhibits proliferation in cells with intact TGFβ signaling than in cells in which the TGFβ type II receptor has been deleted. Rapamycin potentiation of TGFβ-dependent Smad2 phosphorylation may be partially responsible for this effect.

MMTV-Her2/neu transgenic mouse tumors are known to undergo EMT in vivo, which is associated with downregulation of Her2 and upregulation of other receptor tyrosine kinases upon which the post-EMT cells become dependent [23]. Here we found that the post-EMT cells are resistant to rapamycin effects in the presence of 10% FBS and that rapamycin strongly potentiates their HGF-driven proliferation. This effect correlates with increased c-Met and Erk phosphorylation in response to rapamycin co-treatment or pretreatment. Similar effects were observed in the MDA-MB-231 and BT549 human breast cancer cell lines. Rapamycin cooperated with HGF to increase the proliferation of the HCC1954 human breast cancer cell line, but did not produce significant differences in the signaling pathways examined. However, the cooperative induction of the proliferation of HCC1954 cells by rapamycin and HGF requires c-Met because it was reduced by a c-Met tyrosine kinase inhibitor or by shRNA-mediated c-Met knockdown. Further, rapamycin efficacy in the MDA-MB-361 human breast cancer cell line was reduced by forced TPR-Met expression. These results suggest that in some cancers that express c-Met, rapalog treatment might increase rather than decrease tumor growth and aggressive properties.

Rapamycin and the mTORC1/2 inhibitor Torin increased Akt phosphorylation on the PDK1 site Thr^308^ in the rapalog resistant HCT116 colon cancer cell line. Additional studies will be required to determine how common this effect is. Thr^308^ is phosphorylated by PDK1 [26], thus if mTORC1/2 inhibitors stimulated all PDK1-dependent phosphorylation events, most of the AGC family of kinases would become activated. This may indicate the existence of a Thr^308^ kinase other than PDK1, which is activated in response to mTORC1/2 inhibitors. Unfortunately, PDK1 inhibitors may produce confusing results with respect to effects on Akt phosphorylation on Thr^308^ [33], and inadvertently activate Phosphatidylinositol 3-kinase (PI3K) activity [34]. Thr^308^ phosphorylation was enhanced with increasing Torin concentrations, while Torin had a biphasic effect on the levels of IGF1R and IRS1, suggesting that these are two mechanistically distinct effects through which mTOR inhibitors can increase Akt signaling.

Novel mechanisms of cross talk have been revealed which may have bearing on these results. mTORC1 activation of S6K1 followed by Sin1 phosphorylation inhibits mTORC2, preventing Akt activation [35]. Akt phosphorylation is thought to occur in a stepwise manner in which the hydrophobic site, Ser^473^ is first phosphorylated by mTORC2, and this in turn facilitates PDK1 mediated phosphorylation of Thr^308^ (reviewed in [36]). This mechanism however is unlikely to account for the results presented in Fig. 4 because rapamycin would be predicted to increase Akt Thr^308^ phosphorylation more effectively than Torin, while the opposite is observed. A recent study [34] shows that inhibition of multiple AGC-family kinases, as might occur with an mTORC1/2 inhibitor such as Torin, blocks a negative feedback loop resulting in increased expression of PI3K. Such a mechanism could explain the Torin-mediated increase in Akt phosphorylation on the PDK1 site. It should also be noted however that Akt may be activated through other sites in addition to Ser^473^ and Thr^308^ including various tyrosine phosphorylation sites (reviewed in [37]), and a recently described C-terminal site phosphorylated by Cyclin A/Cdk2 that may facilitate or substitute for Ser^473^ phosphorylation [38].

The observation that MEK inhibition partially downregulates rapamycin- and Torin-induced Akt Thr^308^ phosphorylation suggests an important role for cross talk between the MEK/Erk and Akt/mTOR pathways in this effect. The finding that both U0126 and Torin suppress Akt Ser^473^ phosphorylation indicates cross talk between the MEK/Erk and Akt/mTOR pathways in HCT116 cells in the control of this site as well. The observation that inhibition of both of these signaling axes is required to block S6 phosphorylation, but that only inhibition of mTORC1 is required to inhibit S6K phosphorylation, suggests the existence of at least two kinases capable of mediating S6 phosphorylation in these cells. Our findings may provide an explanation for results showing that PI3K and MEK inhibitors exhibit enhanced efficacy in HCT116 and HT29 xenograft tumor studies when used in combination as compared with monotherapy [39].

Thus, the effect of mTOR inhibitors on the proliferation of a given cell will depend on many factors including the condition of the TGFβ signaling pathway and TGFβ levels, the status of the c-Met signaling pathway and HGF levels, in the case of Torin, the concentration of the drug, and finally, cross-talk and functional redundancy between the MEK/Erk and mTORC2/Akt and Akt/mTORC1 signaling axes. Further studies are needed to determine to what extent each of the signaling effects described above influences the efficacy of rapalogs in animal models and in the clinic. Importantly, such observations may explain how in some instances rapalogs are ineffective or, worse, inadvertently promote cancer metastasis [40].
